# Temporal Variation and Statistical Assessment of the *b* Value off the Pacific Coast of Tokachi, Hokkaido, Japan

**DOI:** 10.3390/e21030249

**Published:** 2019-03-05

**Authors:** Weiyun Xie, Katsumi Hattori, Peng Han

**Affiliations:** 1 Graduate School of Science and Engineering, Chiba University, Chiba 263-8522, Japan; 2 Graduate School of Science, Chiba University, Chiba 263-8522, Japan; 3 Center for Environmental Remote Sensing, Chiba University, Chiba 263-8522, Japan; 4 Southern University of Science and Technology, Shenzhen 518055, China

**Keywords:** *b* value, bootstrap, Akaike Information Criterion, off the Pacific coast of Tokachi, Hokkaido

## Abstract

The Gutenberg-Richter Law describes the frequency-magnitude distribution of earthquakes. A number of studies have shown that the slope (*b* value) of the relationship between frequency and magnitude decreased before large earthquakes. In this paper, we investigate the temporal variation of the *b* value off the Pacific coast of Tokachi, Hokkaido, Japan, during 1990–2014. The magnitude of completeness (Mc) in the catalog is evaluated by combining the maximum curvature (MAXC) technique and the bootstrap approach. Then, the *b* value, and its uncertainty, is computed by using the maximum likelihood estimation. The Akaike Information Criterion (AIC) with the bootstrap approach is introduced to statistically assess the temporal variation of *b* values and quantify the significance level. The results show a decrease in trends of the *b* value prior to two large earthquakes (26 September 2003 (M8.0) and 11 September 2008 (M7.1)) in the analyzed area. In addition, the decrease of *b* values shows certain statistical significance three months before the 2003 Earthquake off the Pacific coast of Tokachi (M8.0). It is concluded that the *b* value with statistical assessment may contain potential information for future large earthquake preparation off the Pacific coast of Tokachi, Hokkaido, Japan.

## 1. Introduction

The Gutenberg-Richter (GR) law, $\log_{10}N\left(M\right)=a-bM$ shows the relationship between the magnitude and frequency of earthquake, where *M* is the magnitude, *N* is the cumulative frequency of earthquakes with magnitude larger than or equal to *M*, and *a* and *b* are constants. The *b* value measures differences in the relative proportion of small and large earthquakes. The *b* value is a widely reported seismicity parameter essential for seismic hazard analysis [1]. To date, several studies [2,3,4,5] have demonstrated the possible decrease of the *b* value prior to great earthquakes [6] and claimed that the variation of the *b* value could reflect the physical process of stress evolution and crack growth. Furthermore, recent results based on the natural time analysis of earthquake data [7,8] revealed that a fall of the *b* value observed before large earthquakes reflects an increase of the order parameter fluctuations upon approaching the critical point (main shock) and stems from both origins of self-similarity, i.e., the process increments infinite variance and/or process memory, as discussed in [9]. Experimental study [10] has suggested that the *b* value in rock samples, revealed by detecting cracks in the material under stress, had a long-term decrease trend before the main rupture. Nanjo and Yoshida [11] analyzed the slip-deficit rate (SDR) and the *b* value in the Nankai Trough, Japan, and revealed that the *b* value is inversely correlated with the stress accumulation rate. Stress accumulation may result in the increase in a proportion of larger earthquakes and result in a smaller *b* value. On the contrary, stress release brings a larger *b* value. Therefore, a significant decrease in the *b* value is a sign of a potential impending large rupture. 

Due to the subduction of the Pacific plate off the Pacific coast of Tokachi, Hokkaido, Japan is a seismic active region with several M8 class historical earthquakes [12]. The latest one was the M8.0 earthquake in 2003. In this study, we analyze the catalog in this region from 1990 to 2014 to examine whether there is any indicator of the occurrence of large earthquakes in temporal variation of the *b* value. To make a robust estimation of magnitude of completeness (Mc) in the catalog, the maximum curvature (MAXC) technique [13] is applied in combination with the bootstrap approach [14]. The temporal variation of the *b* value and the significance level is quantitatively assessed using the Akaike Information Criterion (AIC) [15].

## 2. Earthquake Data

**Figure 1:**
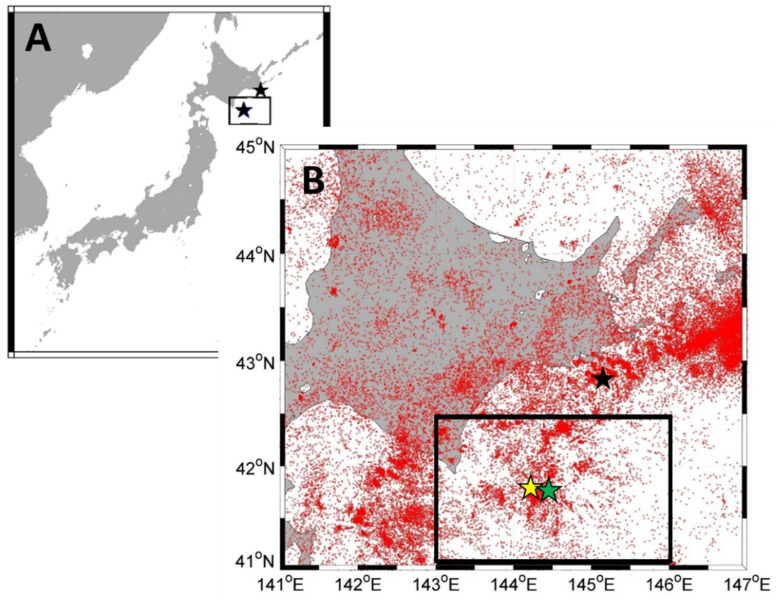
(**A**) Location of the study area. The black rectangle indicates the study region. The black stars inside the rectangle show the epicenters of the 2003 off the Pacific coast of Tokachi earthquake (M8.0) and the 2008 off the Pacific coast of Tokachi earthquake (M7.1). The black star out of the rectangle is the 2004 earthquake off the Pacific coast of Kushiro-oki (M7.1). (**B**) Map of seismicity in Hokkaido, Japan during 1990–2014. The black rectangle is the study area. The black star out of the rectangle is the 2004 earthquake off the Pacific coast of Kushiro-oki (M7.1). The yellow star shows the epicenter of the 2003 earthquake off the Pacific coast of Tokachi (M8.0) and the green one is the 2008 earthquake off the Pacific coast of Tokachi (M7.1).

We used earthquake catalogs issued by the Japan Meteorological Agency (JMA) during the period of 1990–2014 (https://www.data.jma.go.jp/svd/eqev/data/bulletin/shindo.html). The study area is 41° N–42.5° N, 143° E–146° E, as shown in Figure 1. We analyzed the earthquakes with a depth of 0–90 km. The selected area refers to the slip range reported in [16]. In the analyzed period, there were two large earthquakes, with magnitudes greater than 7, which occurred on 26 September 2003 (M8.0) and 11 September 2008 (M7.1). The total number of the earthquake events in the study area was 23,954 during 1990–2014. 

## 3. Methods

### 3.1. Estimation of Magnitude of Completeness (Mc)

Because of limitations in the detection capability of the seismograph network, weak earthquakes are not recorded completely, which leads to the practical deviation of frequency-magnitude distribution (FMD). Therefore, it is of key importance to discover the magnitude of completeness (Mc) in the earthquake catalog for the *b* value computation. However, the detection capability varies over time, and the Mc parameter changes accordingly. So far, several methods for Mc estimation, such as the Goodness-of-Fit Test (GFT) [17], the Entire Magnitude Range (EMR) [18], the Maximum Curvature (MAXC) [13], etc., have been proposed. Here we use the MAXC technique for estimation of the Mc, as MAXC is a robust and simple method to estimate Mc by finding the magnitude bin with the highest frequency of events in the FMD plot.

In order to reveal the Mc variation over time, we compute Mc using a moving time window of 500 event samples and a 50 event shifting step. We estimate the confidence limit of the Mc results in each window using the bootstrapping approach proposed by [14]. In this approach, we randomly sample a dataset of 500 events out of the original 500 earthquake samples and then use the maximum curvature (MAXC) technique to compute the Mc of the resampled dataset. We repeat this 1000 times and take the mean value to represent the Mc of the window and the standard deviation as Mc errors. 

### 3.2. Calculation of the b Value

In this study, the maximum likelihood method is used to compute the *b* value [19,20]:

(1) $$b=\frac{1}{\ln\left(10\right)\left(\bar{M}-M_{C}\right)}$$

where $\bar{M}$ denotes the average magnitude of a group of earthquakes with *M* ≥ *M*_c_, and *M*_c_ is the magnitude of completeness. We estimate the confidence limit of estimated *b* value using the analytic approach given by [19]:

(2) $$\sigma_{b}=\frac{b}{\sqrt{N}}$$

where *N* is the total number of events of the given sample. 

### 3.3. Statistical Assessment of Temporal b Value Changes

To quantitatively assess the differences between the anomalous *b* value changes over time, we perform statistical test proposed by [21]. In this method, *P_b_* is defined as the probability of the hypothesis that the *b* values of two sample time windows come from the same population. The P_b_ is derived from the AIC (Akaike Information Criterion) [15]. Comparing the AIC_0_ for two samples with the same *b* value (*b*_0_) and the AIC_12_ for two catalogs with different *b* values (*b*_1_ and *b*_2_) leads to the difference ΔAIC:

(3) $$\Delta \mathrm{AIC}=-2\left(N_{1}+N_{2}\right)\ln\left(N_{1}+N_{2}\right)+2N_{1}\ln\left(N_{1}+\frac{N_{2}b_{1}}{b_{2}}\right)+2N_{2}\ln\left(N_{2}+\frac{N_{1}b_{2}}{b_{1}}\right)-2$$

where *N*_1_ and *N*_2_ are the number of events in each sample and *b*_1_ and *b*_2_ are the *b* value of each sample time window. The probability *P_b_* that the *b* values are not different is given by [22]:

(4) $$P_{b}=e^{\left(-\Delta AIC/2\right)-2}$$

Utsu [20] defined the difference between two *b* values as insignificant when ΔAIC < 2 ($P_{b}\approx 0.05$). The difference is considered highly significant for ΔAIC > 5 (with a corresponding probability of $P_{b}\approx 0.01$).

In practical terms, to determine whether the *b* value changes at a given time point is significant or not, we must determine an appreciate reference to compute the ΔAIC. As an attempt, we took the earthquakes in the first five years of the analyzed period as the reference, because the *b* value variation there was relatively stable and not affected by aftershocks very much. To avoid bias caused by reference selection, we applied the bootstrap approach again by randomly sampling *N*_2_ events from the first five years of earthquake catalogs and computed ΔAIC values between the target window (N_1_ events) and resampled reference window (*N*_2_ events). We repeated this 1000 times and counted the percentage for ΔAIC ≥ 2, denoted by *P*. A larger *P* value indicates a higher significance level of *b* value changes at the target time window compared with reference period, and vice versa. By this mean, we can quantify temporal changes of the *b* value using *P* parameter. 

## 4. Results

**Figure 2:**
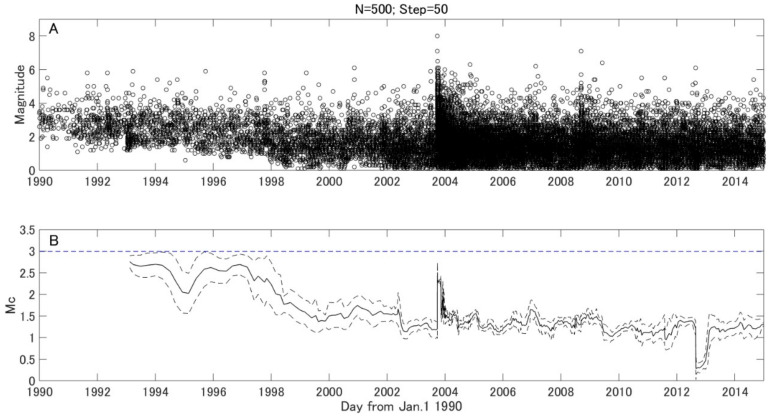
Earthquake distribution and magnitude of completeness (Mc) variation. (**A**) Temporal distribution of earthquakes in the study area from 1990 to 2014. (**B**) A solid line indicates the temporal variation of Mc, and broken lines show the error of Mc (standard deviation estimated by bootstrap method). The window length *N* is 500 earthquakes, and the step is 50 earthquakes. The blue line shows the Mc adopted in this study.

The completeness of the earthquake catalog for the earthquake off the Pacific coast of the Tokachi region during 1990–2014 was investigated by the MAXC method. Figure 2 shows the temporal variation of the Mc derived by a moving window of 500 samples, with a 50 samples step. It was found that the Mc is relatively large because of the weak detection capability in 1990s. Because a dense seismograph network with high sensitivity was gradually built after the 1995 Kobe earthquake [23], the Mc gradually decreased in late 1990s. A notable increase of Mc can be observed right after the 2003 earthquake off the Pacific coast of Tokachi (M8.0). This is because smaller aftershocks are commonly missed in the catalog either due to a stronger background noise induced by previous shocks or the overlaying of seismic waves of aftershocks occurring within a short time span [24]. From Equation (1) we find that the *b* value depends on Mc selection. To avoid the influence of the Mc, it is better to fix the Mc during the whole analyzing period [5]. As shown in Figure 2B, all Mcs are smaller than 3.0. Therefore, the earthquake catalog in the off the Pacific coast of Tokachi region during 1990–2014 is complete for earthquakes M ≥ 3.0. We chose Mc = 3.0 for the *b* value computation in this study. There were 2369 earthquakes with magnitude larger or equal to the Mc in the study area during 1990–2014. 

**Figure 3:**
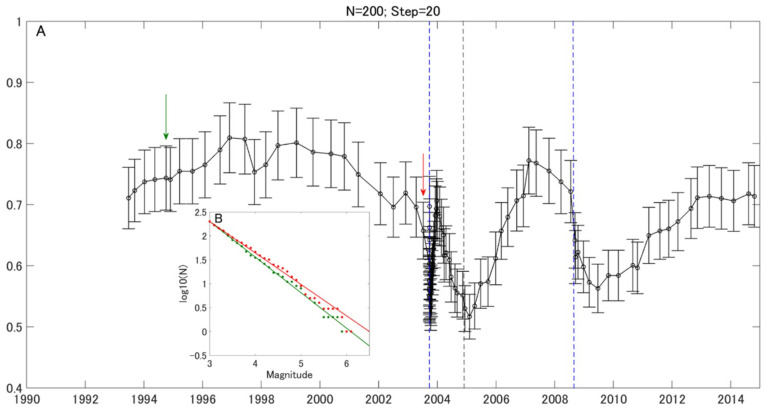
Temporal variation of *b* values. (**A**) A black line shows the temporal variation of *b* values during 1992–2014 for the window length *N* = 200, Step = 20. The vertical blue lines show the time of the 2003 earthquake off the Pacific coast of Tokachi (M8.0) and the 2008 earthquake off the Pacific coast of Tokachi (M7.1). The vertical grey line show the 2004 earthquake off the Pacific coast of Kushiro (M7.1), which is close to the study area. (**B**) The frequency-magnitude distribution of earthquakes in the windows is indicated by arrows with corresponding color in (**A**).

Figure 3 shows the temporal variation of *b* values. In this computation, the window length and the step were set as 200 earthquakes and 20 earthquakes, respectively. The two vertical dashed blue lines show the 2003 (M8.0) and the 2008 (M7.1) earthquakes off the Pacific coast of Tokachi. The *b* value, during 1993–1997, shows an increasing trend, which then gradually decreases until the occurrence of the 2003 M 8.0 earthquake. After the M8.0 earthquake, the *b* value increased quickly and then decreased again in 2004, when an M7.1 earthquake (29 November 2004, 42.9 °N, 145.3 °E, depth 50 km) occurred close to the study area. From 2005 to the beginning of 2007, the *b* value recovered. Then, it began to decrease, and the 2008 M7.1 Tokachi earthquake occurred. After that earthquake, the *b* value gradually increased to the initial value of the whole analyzed period.

**Figure 4:**
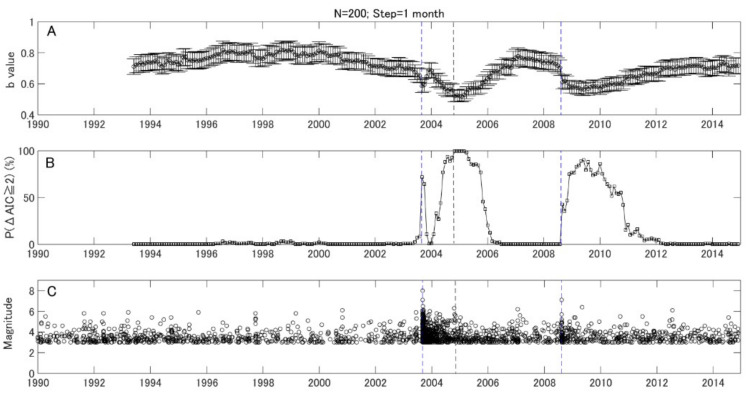
The monthly variations of *b* values, *P* values, and earthquakes in the study area. The vertical blue lines show the time of the 2003 off the Pacific coast of Tokachi earthquake (M8.0) and the 2008 earthquake off the Pacific coast of Tokachi (M7.1). The vertical grey line show the 2004 earthquake off the Pacific coast of Kushiro-oki (M7.1) which is close to the study area. (**A**) The monthly variation of *b* values with *N* = 200. The step of shifting window is 1 month. (**B**) The temporal variation of *P* (ΔAIC ≥ 2) value. (**C**) The temporal distribution of earthquakes with M ≥ Mc.

**Figure 5:**
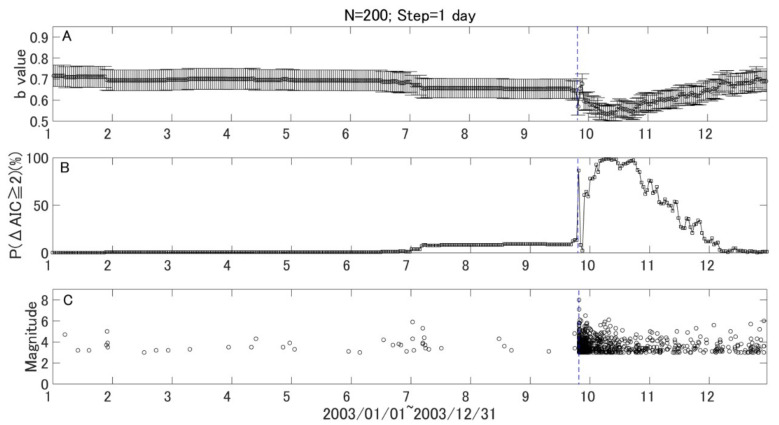
Daily variations of *b* values, *P* values, and earthquakes in 2003. The vertical blue line show the time of the 2003 off the Pacific coast of Tokachi earthquake (M8.0). (**A**) Daily variation of *b* values with a window of 200 earthquakes and time step of one day, (**B**) Daily variation of *P* (ΔAIC ≥ 2) values, and (**C**) the temporal distribution of earthquake events. The horizontal axes are the sequential months in 2003.

**Figure 6:**
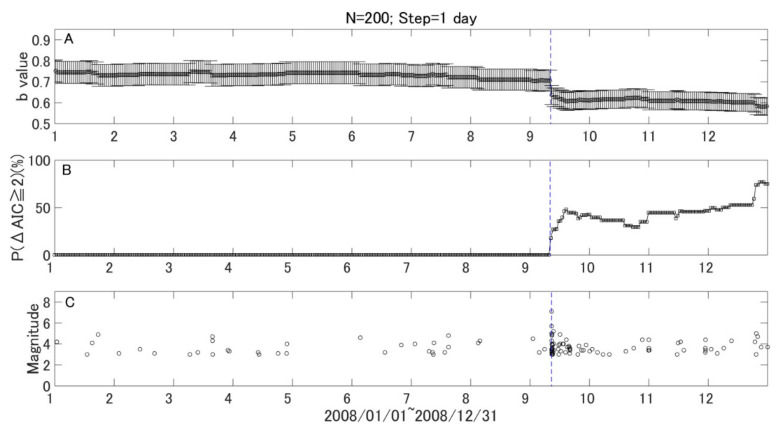
Daily variations of *b* values, *P* values, and earthquakes in 2008. The vertical blue line show the time of the 2008 off the Pacific coast of Tokachi earthquake (M7.1). (**A**) Daily variation of *b* values with a window of 200 earthquakes and time step of one day, (**B**) Daily variation of P (ΔAIC ≥ 2) values, and (**C**) the temporal distribution of earthquake events. The horizontal axes are the sequential months in 2008.

To watch the *b* value evolution with time more closely, we kept the window length of 200 samples and reset the step to one month. A backward window was adopted as it enables one to examine *b* values at the target time point without using future earthquake data. We further assessed the temporal change of *b* values quantitatively using *P* values. Figure 4A shows the results of temporal variation with a one-month step. The monthly variation of the *b* values shows a similar pattern with the variation shown in Figure 3, while the variation after the 2003 M8.1 earthquake in the monthly variation is much clearer. Figure 4B gives the statistical significance of the *b* value change in terms of the P parameter, which counts the percentage for ΔAIC ≥ 2. Here, the sample size in the target window (*N*_1_) and resampled reference window (*N*_2_) are both 200. It is found that the *P* parameter is relatively stable, and most of the high values appeared during aftershock period. However, there was a small but notable increase a few months before the 2003 Tokachi earthquake. To confirm this change, we reset the step to one day and kept the window size as 200 events. The results are given in Figure 5 and Figure 6 for the 2003 M8.0 and 2008 M7.1 earthquakes, respectively. It was found that the *P* value increased from background about 1.28 to 12.42 just three months before the 2003 M8.1 Tokachi earthquake. However, no similar change was found before the 2008 M7.1 earthquake.

## 5. Discussion

Mc is of essential importance in analyzing earthquake catalogs. The traditional method for Mc estimation is to calculate Mc over the entire analyzed period. Ignoring that the temporal variation of Mc may result in the underestimation of Mc in the beginning of the catalog, because most seismic observation networks improve with time. In this study, we checked the Mc variation and its error by employing the bootstrap approach. This enabled us to obtain Mc robustly. So far, a number of methods have been proposed to determine Mc. We selected the MAXC method because it is simple and wildly used [25]. Indeed, [26] comparing the EMR method with other three existing techniques (including GFT and MAXC) finds that EMR shows a superior performance when applied to synthetic test cases or real data from regional and global earthquake catalogues. This method, however, is also the most computationally intensive. The Mcs estimated by EMR, GFT, and MAXC are quite similar, with an average difference of around 0.1 for synthetic catalogs and 0.2 for real catalogs [23]. In Figure 2B, the maximum Mc is 2.7; therefore, earthquakes with M ≥ 3.0 are complete, suggesting that the temporal *b* value changes were not likely caused by Mc estimation.

In Figure 3 and Figure 4, there is a drop after the two large events. These brief dips in the *b* value variation are due to more moderate-sized events induced by dynamic stress changes during the aftershock period, a feature common to most aftershock sequences [1]. Nevertheless, there was a recovery phase, which was not observed in the 2008 M7.1 case, after the 2003 M8.0 earthquake. The *b* value recovered after the 2003 M8.0 earthquake, because the tress was released during the large event. The *b* value decreased again in 2004, which may relate to the stress rebuilding before the 2004 M7.1 event close to the study region in Hokkaido. As for the 2008 M7.1 event, the *b* value decreased after the event, and a low value persisted until 2011. This result may be because the stress was not released largely until the onset of the 2011 Tohoku M9.0 earthquake. The differences of the *b* value variation after the 2003 M8.0 and 2008 M7.1 events may imply different stress evolution processes. 

Like before the two earthquakes, there is a clear decreasing trend, even though the duration of the trend before the 2008 M7.1 event was not long. These decreasing trends of the *b* value are consistent with those obtained before the 2008 Wenchuan (M8.0) [5] and the 2011 Tohoku (M9.0) [4] events. The *b* value began to decrease about 20 years prior to the 2011 M9.0 earthquake in the whole rupture region and about seven years prior to the M9.0 event in the Tohoku area, where the largest slip happened [5]. For the Wenchuan case, the *b* value began to decrease about 6.5 years prior to the main shock in the rupture zone [4]. In this study, we found that the *b* value began to decrease about 6.7 years before the 2003 M8.0 event and about 1.7 years before the 2008 M7.1 event. The durations of *b* value decrease before the onset of the M8.0 Wenchuan earthquake and the 2003 M8.0 earthquake in Hokkaido are quite similar, possibly due to their same magnitude. As we can see, there seems to be a correlation between duration of a *b* value decrease before main shock and its magnitude, which might be useful for larger earthquake forecasting. More case studies are required to clarify the details of this relationship.

Previous studies have suggested that there is a correlation between global earthquake rates and sunspot cycles [27,28,29]. In order to examine whether the long-term *b* value variation observed in Hokkaido is a local phenomenon governed by regional stress evolution or a global phenomenon related to solar activities, we performed a test in the Tokai area, which is far from Hokkaido. We analyzed the seismicity there during the same period and computed the *b* value using the same technique. We found that the *b* value variations were quite different from those in Hokkaido. The correlation coefficient between the *b* values in the Hokkaido and Tokai regions during 2000–2005 was 0.268, suggesting that the *b* value obtained in this study has a local character and reflects stress evolution in the Hokkaido region. 

It is interesting to find a similar decrease of *b* values at large distances for Hokkaido and Sumatra around the year 2000. Some previous studies indicate that there is a correlation between space weather and seismicity: there is a higher seismicity during the rising and the decay of the solar cycle [27,28]. One may think the decrease trend of the *b* value is a global effect associated with solar activities. However, the *b* value is not the seismicity rate. The *b* value shows the relationship between the magnitude and frequency of earthquake, and measures differences in the relative proportion of small and large earthquakes. A higher seismicity rate does not imply a higher b value or lower *b* value. Even though there is a correlation between the seismicity rate and solar cycles, it does not mean that the *b* value should be correlated with solar cycles. Moreover, if solar cycles influence global seismicity and then affect the *b* value, then the *b* value at a global scale should be correlated with solar cycles. In fact, the *b* value at a global scale is quite stable and close to the constant value of 1.0 at long time scale [29,30]. In a short time-span, the *b* value could be lower or higher than solar cycles, depending on the dominate focal mechanism of the vents in the analyzed period. To make a close investigation on the relation between global *b* value changes and solar activities, we analyzed the *b* values for global events with *M* ≥ 5.5 and computed the Person correlation coefficient between the *b* values and yearly mean total sunspot numbers from 2000–2018. The coefficient is 0.0616, suggesting almost no correlation between the two. On the other hand, if the *b* value decrease is a global phenomenon related to solar cycles, then the decrease trend should end at a similar time. However, as we showed before, the decrease trend of the *b* value for *M* 8.0 earthquakes ended just after the onset of the large events in Wenchuan, Hokkaido, and Tohoku in 2003, 2008, and 2011, respectively. The different trend change time of the *b* values indicates that the *b* values at these regions were not controlled by one global factor, but by several regional factors, such as the regional stress levels.

To date, most studies on *b* values focus on their temporal and/or spatial variations. The changes in *b* values have been analyzed qualitatively, rather than quantitatively. In order to make use of the *b* value in practical earthquake forecasts, we must quantify the variation. The method proposed by [18], derived from AIC, can then be introduced to compare the *b* value between the target window and reference, quantitatively. Another important thing is how to find an appropriate reference. In this study, as an attempt, we chose the first five years as a reference period and employed the bootstrap method to select reference samples. By combing Utsu’s method and the bootstrap method, we developed a robust parameter *P* to quantify *b* value change in each time window. As one can see in Figure 4B and Figure 5B, the *P* value is quite stable and increased just three months prior to the 2003 M8.0 event. If we ignore the aftershock period, this increase is quite unique. Therefore, compared to the temporal variation of *b* values, the *P* value, which is usually stable, might have a potential advantage in mid-term (a few months) earthquake forecast. However, more case studies are needed to test the *P* parameter to see if it is an effective indicator for large earthquake occurrences.

## 6. Conclusions

In this study, we investigated the temporal variation of the *b* value off the Pacific coast of Tokachi, Hokkaido, Japan, during 1990–2014. The magnitude of completeness (Mc) in the catalog was evaluated by combining the maximum curvature (MAXC) technique and the bootstrap approach. The *P* parameter, which counts the percentage for ΔAIC ≥ 2, was introduced to quantify the significance level and statistically assess the temporal variation of the *b* value. The results show clear decreasing trends of the *b* value prior to two major large earthquakes (26 September 26 2003 (M8.0) and 11 September 2008 (M7.1)) in the analyzed area. In addition, the *P* parameter shows a clear increase three months before the 2003 earthquake off the Pacific coast of Tokachi (M8.0). It is concluded that the *b* value with statistical assessment may contain potential information for future large earthquake preparation off the Pacific coast of Tokachi, Hokkaido, Japan.

## References

[1] Wyss M., Wiemer S. (2000). Change in the probability for earthquakes in southern California due to the Landers magnitude 7.3 earthquake. Science.

[2] Scholz C.H. (1968). The frequency-magnitude relation of micro fracturing in rock and its relation to earthquakes. Bull. Seism. Soc. Am..

[3] Sahu O., Saikia M. (1994). The *b* value before the 6th August, 1988 India-Myanmar border region earthquake—A case study. Tectonophysics.

[4] Nanjo K., Hirata N., Obara K., Kasahara K. (2012). Decade-scale decrease in *b* value prior to the M9-class 2011 Tohoku and 2004 Sumatra quakes. Geophys. Res. Lett..

[5] Shi H., Meng L., Zhang X., Chang Y., Yang Z., Xie W., Hattori K., Han P. (2018). Decrease in *b* value prior to the Wenchuan earthquake (M (s) 8.0). Chin. J. Geophys..

[6] Main I., Meredith P., Jones C. (1989). A reinterpretation of the precursory seismic b-value anomaly from fracture mechanics. Geophys. J. Int..

[7] Varotsos P.A., Sarlis N.V., Skordas E.S., Lazaridou M.S. (2013). Seismic electric signals: An additional fact showing their physical interconnection with seismicity. Tectonophysics.

[8] Sarlis N.V., Skordas E.S., Varotsos P.A., Nagao T., Kamogawa M., Tanaka H., Uyeda S. (2013). Minimum of the order parameter fluctuations of seismicity before major earthquakes in Japan. Proc. Natl. Acad. Sci. USA.

[9] Varotsos P.A., Sarlis N.V., Skordas E.S. (2012). Order parameter fluctuations in natural time and b-value variation before large earthquakes. Nat. Hazards Earth Syst. Sci..

[10] Lei X., Kusunose K., Nishizawa O., Cho A., Satoh T. (2000). On the spatio-temporal distribution of acoustic emissions in two granitic rocks under triaxial compression: The role of pre-existing cracks. Geophys. Res. Lett..

[11] Nanjo K., Yoshida A. (2018). A b map implying the first eastern rupture of the Nankai Trough earthquakes. Nat. Commun..

[12] Utsu T. (1972). Large Earthquakes near Hokkaido and the Expectancy of the Occurrence of a Large Earthquake off Nemuro.

[13] Wiemer S., Wyss M. (2000). Minimum Magnitude of Completeness in Earthquake Catalogs: Examples from Alaska, the Western United States and Japan. Bull. Seism. Soc. Am..

[14] Schorlemmer D., Neri G., Wiemer S., Mostaccio A. (2003). Stability and significance tests for b-value anomalies: Example from the Tyrrhenian Sea. Geophys. Res. Lett..

[15] Akaike H. (1974). A new look at the statistical model identification. IEEE Trans. Autom. Control.

[16] Miyazaki S., Larson K., Choi K., Hikima K., Koketsu K., Bodin P., Haase J., Emore G., Yamagiwa A. (2004). Modeling the rupture process of the 2003 September 25 Tokachi-Oki (Hokkaido) earthquake using 1 Hz GPS data. Geophys. Res. Lett..

[17] Cao A., Gao S. (2002). Temporal variations of seismic b-values beneath northeastern japan island arc. Geophys. Res. Lett..

[18] Ogata Y., Katsura K. (1993). Analysis of temporal and spatial heterogeneity of magnitude frequency distribution inferred from earthquake catalogs. Geophys. J. Int..

[19] Aki K. (1965). Maximum likelihood estimate of b in the formula log N = a − bM and its confidence limits. Bull. Earthq. Res. Inst..

[20] Utsu T. (1965). A method for determining the value of b in a formula log n = a − bM showing the magnitude-frequency relation for earthquakes. Geophys. Bull. Hokkaido Univ..

[21] Utsu T. (1992). On seismicity. Report of the Joint Research Institute for Statistical Mathematics.

[22] Utsu T. (1999). Representation and analysis of the earthquake size distribution: A historical review and some new approaches. Pure Appl. Geophys..

[23] Obara K., Kasahara K., Hori S., Okada Y. (2005). A densely distributed high-sensitivity seismograph network in japan: Hi-net by national research institute for earth science and disaster prevention. Rev. Sci. Instrum..

[24] Zhuang J., Ogata Y., Wang T. (2017). Data completeness of the Kumamoto earthquake sequence in the JMA catalog and its influence on the estimation of the ETAS parameters. EarthPlanets Space.

[25] Mousavi S., Mariotti R., Bagnoli F., Costantini L., Cultrera N.G., Arzani K., Pandolfi S., Vendramin G.G., Torkzaban B., Hosseini-Mazinani M. (2017). The eastern part of the Fertile Crescent concealed an unexpected route of olive (*Olea europaea* L.) differentiation. Ann. Bot..

[26] Woessner J., Wiemer S. (2005). Assessing the quality of earthquake catalogues: Estimating the magnitude of completeness and its uncertainty. Bull. Seism. Soc. Am..

[27] Shestopalov I.P., Kharin E.P. (2014). Relationship between solar activity and global seismicity and neutrons of terrestrial origin. Russ. J. Earth Sci..

[28] Rajesh R., Tiwari R.K. (2014). Brief Communication: Correlation of global earthquake rates with temperature and sunspot cycle. Nat. Hazards Earth Syst. Sci. Discuss..

[29] Kagan Y.Y. (1999). Universality of the seismic moment-frequency relation. Seismicity Patterns, Their Statistical Significance and Physical Meaning.

[30] Frohlich C., Davis S.D. (1993). Teleseismic *b* values; or, much ado about 1.0. J. Geophys. Res. Solid Earth.

